# Supplementary material for: Cryopreservation Differentially Alters the Proteome of Epididymal and Ejaculated Pig Spermatozoa
Source: Int J Mol Sci. 2019 Apr 11;20(7):1791. doi: 10.3390/ijms20071791 (PMC6479301; doi:10.3390/ijms20071791)
Supplement: Supplementary file 1 [file ijms-20-01791-s001.zip › Figure S2. Cytometry work plan.docx]

A. B.


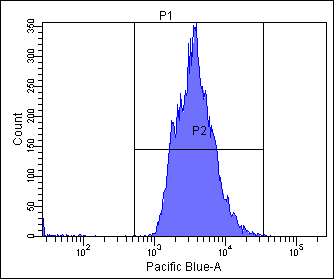

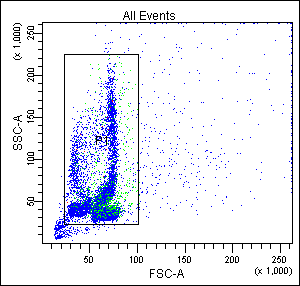


C. D.


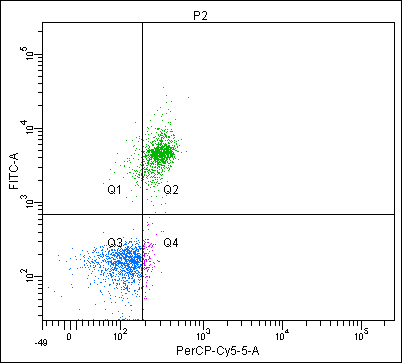

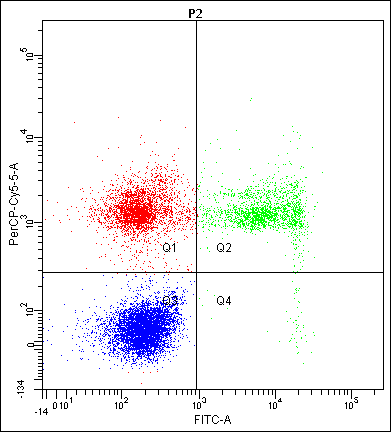


E.


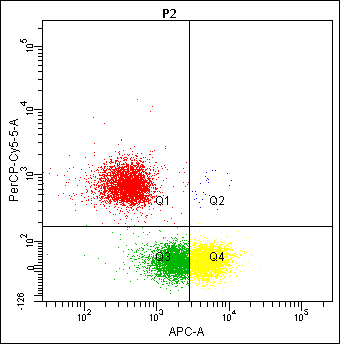


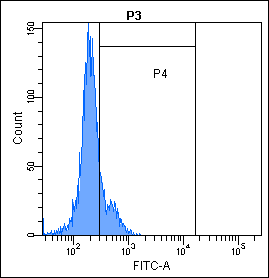

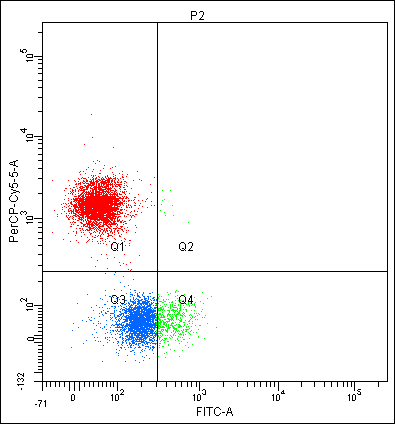
F.

**Flow cytometric gating strategy applied during assessment of the quality and functionality of frozen-thawed pig spermatozoa**. **(A)** FSC-SSC dot plot used to exclude debris; **(B)** Histogram showing events stained with Hoechst 33342 (H-42) used to define sperm populations (P2) based on positive fluorescence to H-42; **(C)** Dot plot used to determine the percentage of viable spermatozoa (region Q3: H-42+/PI(PerCP-Cy5-5-A)-/PNA-FITC-); **(D)** Dot plot used to determine the percentage of viable spermatozoa with high plasma membrane fluidity [region Q4: [H-42+/Yo-Pro-1 (FITC)-]/M-540 (PerCP-Cy5-5-A)+]; **(E)** Dot plot used to determine the percentage of viable spermatozoa with high mitochondrial membrane potential [region Q4: H-42+/PI(PerCP-Cy5-5-A)-/Mitotracker Deep Red 633 (APC-A)+]; **(F)** Dot plot and histogram depicting determination of intracellular hydrogen peroxide (H_2_O_2_) generation. Mean fluorescence intensity of oxidized H_2_DCFDA in the viable sperm population [region Q4: H-42+/PI(PerCP-Cy5-5-A)-/DCF (FITC)+] was measured in the histogram (mean fluorescence intensity of P4 region) and expressed as fluorescence units (FU) per 10^6^ viable spermatozoa.
