# Supplementary material for: Cryopreservation Differentially Alters the Proteome of Epididymal and Ejaculated Pig Spermatozoa
Source: Int J Mol Sci. 2019 Apr 11;20(7):1791. doi: 10.3390/ijms20071791 (PMC6479301; doi:10.3390/ijms20071791)
Supplement: Supplementary file 1 [file ijms-20-01791-s001.zip › Supplementary data/Table S1.docx]

**Table 1.** Quality parameters (mean±SEM) of fresh pig spermatozoa collected from cauda epididymis and ejaculate (10 boars).

| **Sperm parameters** | **Sperm source** | |
| --- | --- | --- |
|  | Cauda epididymis | Ejaculate |
| Total motility (%) | 81.00±1.14 | 81.70±0.83 |
| Progressive motility (%) | 42.40±1.52 | 46.07±1.36 |
| Viability (%) | 86.78±0.86 | 88.51±0.52 |
| Normal morphology^1^ (%) | 95.70±0.56 | 96.37±0.31 |

^1^Excluding the cytoplasmic droplets that were present in 40-55% of the cauda epididymal spermatozoa (6-12% with proximal droplets and 32-48% with distal droplets)
