# Supplementary figures and images for: Cryopreservation Differentially Alters the Proteome of Epididymal and Ejaculated Pig Spermatozoa
Source: Int J Mol Sci. 2019 Apr 11;20(7):1791. doi: 10.3390/ijms20071791 (PMC6479301; doi:10.3390/ijms20071791)

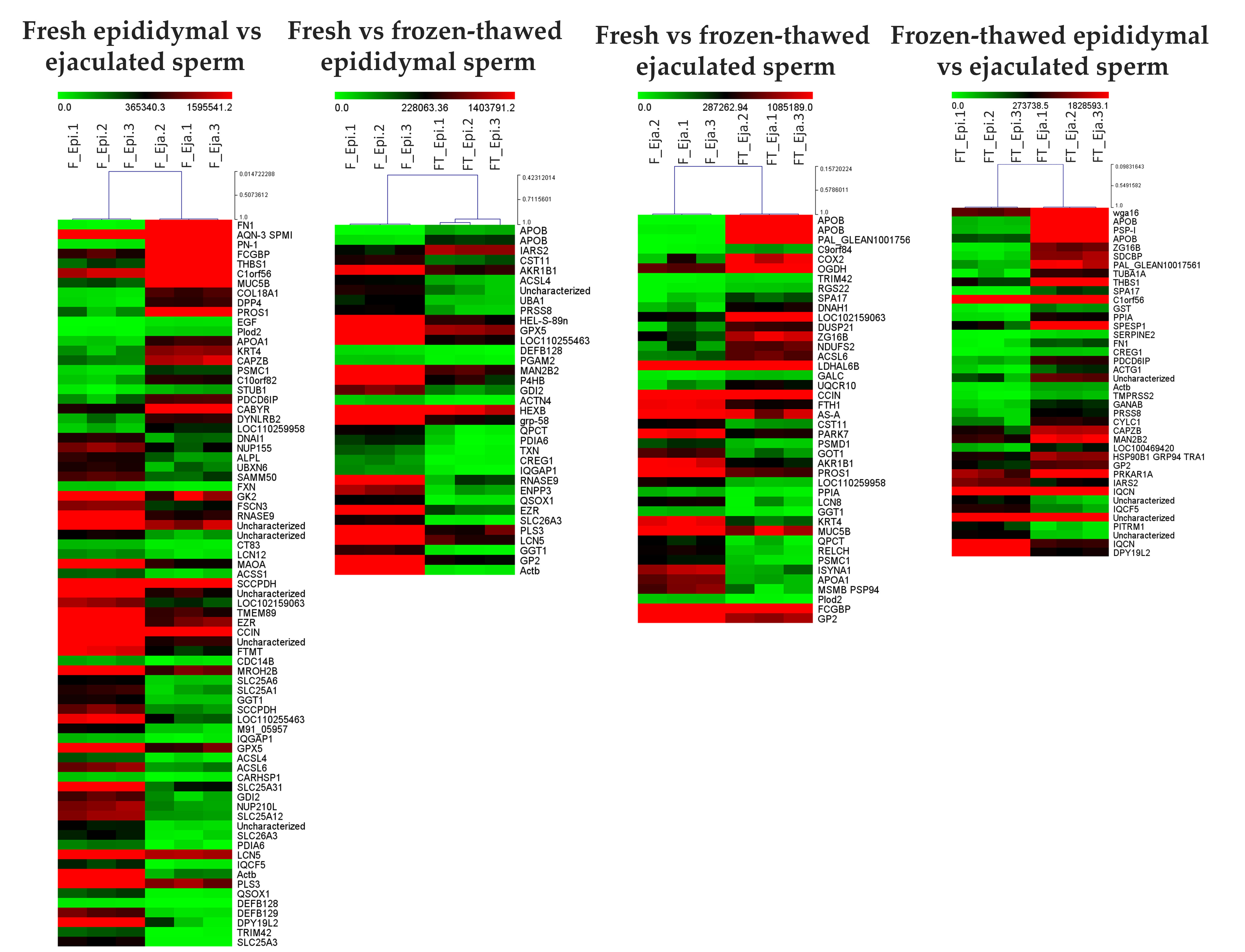

Supplement: Supplementary file 1 [file ijms-20-01791-s001.zip › Supplementary data/Figure S1.jpg]
